# S. AUREUS IS ASSOCIATED WITH A GREATER NEED FOR REOPERATION IN SEPTIC ARTHRITIS OF THE KNEE

**DOI:** 10.1590/1413-785220233102e260592

**Published:** 2023-05-01

**Authors:** LUCAS SAADE FERNANDES, ALEXANDRE JOJI YAGI, ALFREDO DOS SANTOS NETTO, MAURO JOSÉ SALLES, VICTOR MARQUES DE OLIVEIRA, RICARDO DE PAULA LEITE CURY

**Affiliations:** 1 Santa Casa de Misericórdia de Sao Paulo, Faculdade de Ciências Médicas, Departamento de Ortopedia e Traumatologia, Sao Paulo, SP, Brazil.; 2 Santa Casa de Misericórdia de Sao Paulo, Departamento de Ortopedia e Traumatologia, Grupo de Joelho, Sao Paulo, SP, Brazil.; 3 Santa Casa de Misericórdia de Sao Paulo, Faculdade de Ciências Médicas, Sao Paulo, SP, Brazil.; 4 Santa Casa de Misericórdia de Sao Paulo, Faculdade de Ciências Médicas, Departamento de Medicina, Sao Paulo, SP, Brazil.; 5 Universidade Federal de Sao Paulo, Escola Paulista de Medicina, Grupo de Infecção Musculoesquelética, Sao Paulo, SP, Brazil.

**Keywords:** Arthritis, Infectious. Knee. *Staphylococcus aureus*. Infections, Artrite Infecciosa, Joelho, Staphylococcus aureus, Infecções

## Abstract

**Objective:**

To determine the frequency of reoperations in the treatment of adult patients diagnosed with septic arthritis of the knee, the average number of debridements needed to control the infection, the mortality rate, and to assess factors associated with a greater need for reoperation and mortality.

**Methods:**

Retrospective cohort study evaluating 38 adult patients diagnosed with septic arthritis who underwent arthrotomy via a medial parapatellar approach for joint cleaning and debridement. Demographic, clinical, surgical, and microbiological variables of the cases were analyzed by review of medical records. Tests for equality of two proportions, chi-square, and multivariate logistic regression analysis were performed, defining a significance level at 0.05, with 95% confidence interval.

**Results:**

A total of 50% of the cases underwent reoperation, with an average number of required debridement of 2.02 and a mortality rate of 10.5%. Patients with infection caused by Staphylococcus aureus were more likely to need a reoperation compared to patients with positive cultures for other agents (OR 6.0).

**Conclusion:**

In 50% of cases, an average of 2.02 debridements were necessary and the mortality rate was 10.5%. Staphylococcus aureus infection is associated with a 6 times greater chance of additional surgeries. /***Level of Evidence IV, Case Series.***

## RESUMO

**Objetivo:**

Determinar a frequência de reabordagens cirúrgicas no tratamento de pacientes adultos com diagnóstico de artrite séptica do joelho, a média de desbridamentos necessários para o controle da infecção e a taxa de mortalidade, assim como avaliar os fatores associados à maior necessidade de reabordagem e mortalidade.

**Métodos:**

Estudo retrospectivo tipo coorte que avaliou 38 pacientes adultos com diagnóstico de artrite séptica submetidos à artrotomia por via parapatelar medial para limpeza e desbridamento da articulação. Foram analisadas variáveis demográficas, clínicas, cirúrgicas e microbiológicas dos casos, por revisão de prontuário médico, e realizados testes de igualdade de duas proporções, qui-quadrado e análise multivariada de regressão logística, sendo definido um nível de significância de 0,05, com 95% de confiança estatística.

**Resultados:**

50% dos casos foram submetidos à reabordagem cirúrgica, sendo a média de desbridamentos necessários de 2,02 e a taxa de mortalidade de 10,5%. Pacientes com infecção causada por Staphylococcus aureus apresentaram mais chances de necessitar de reabordagem quando comparados aos pacientes com culturas positivas para outros agentes (OR 6,0).

**Conclusão:**

Em 50% dos casos foram necessários, em média, 2,02 desbridamentos e a taxa de mortalidade foi de 10,5%. A infecção por Staphylococcus aureus está associada com seis vezes mais chances de cirurgias adicionais. **Nível de Evidência IV, Série de Casos.**

## INTRODUCTION

Septic arthritis of the knee is a frequent condition and is associated with high morbidity, since the delay in the diagnosis and treatment of infection can lead to irreversible joint damage, with loss of permanent function and even death due to the spread of the infectious process.^1^ ^), (^ ^2^

Septic arthritis enters into the differential diagnoses of patients admitted to the emergency department with pain, joint effusion, and local heat in the knees, which includes osteoarthritis, rheumatoid arthritis, gouty arthritis, other nonspecific arthritis, and septic arthritis itself. ^(^ ^3^ As the treatment of infectious arthritis is based on cleansing and debridement of the joint, in addition to antibiotic therapy, early diagnosis is essential. ^(^ ^4^ Arthrocentesis should be performed for cytological and biochemical evaluation of the synovial fluid, in addition to microbiological analysis with bacterioscopy and culture. ^(^ ^5^ Since culture results are not obtained in the emergency room, and positivity is low even during infection bacterioscopy, cytological evaluation of the synovial fluid is the most important test in the emergency evaluation of these patients. ^(^ ^6^ A synovial fluid white cell count greater than 50,000 cells/mm^3^ is the cutoff number most often used for the diagnosis of septic arthritis. ^(^ ^6^ ^), (^ ^7^ ^), (^ ^8^ ^), (^ ^9^ ^), (^ ^10^

The best surgical treatment technique for septic arthritis of the knee is still controversial. ^(^ ^4^ ^), (^ ^6^ Options include serial aspirations with needles, cleaning, and open debridement (arthrotomy) or arthroscopic. ^(^ ^4^ The superiority of any of the methods has not yet been stablished in the literature, but there is a current tendency to clean and debridement by arthroscopic approach as it allows a wide access to the joint, performs an adequate debridement of synovia, and dilute inflammatory enzymes during the intraoperative period. ^(^ ^4^ Even so, it is often necessary to perform more than one cleansing and debridement of the joint to obtain healing of the infectious process. ^(^ ^5^

The presence of comorbidities associated with immunosuppression, *Staphylococcus aureus* infection, and high cellularity in the synovial fluid are some of the factors described as having a worse prognosis in septic arthritis of the knee, being related to a greater need for surgical reoperations to control the infectious process, but without consensus in the literature. ^(^ ^11^ ^)- (^ ^14^

Thus, this study aims to evaluate the frequency of the need for surgical reoperation to new debridement in the treatment of septic arthritis of the knee of adult individuals, the average debridement necessary to control the infection, the mortality rate of cases, and possible risk factors for greater need for reoperation or higher risk of mortality.

## METHODS

This is a retrospective cohort study that, after approval by the Ethics Committee on Research in Human Beings of the Irmandade da Santa Casa de Misericórdia de São Paulo (CAAE: 45469121.0.0000.5479), evaluated the cases of patients diagnosed with septic arthritis of the knee treated surgically in the Department of Orthopedics and Traumatology of the *Santa Casa de Misericórdia de São Paulo* from May 2015 to January 2021.

The diagnosis of septic arthritis was suspected in clinical and laboratory evaluation, and confirmed by analysis of the synovial fluid. Inclusion criteria were patients with clinically suspected septic arthritis of the knee (knee with acute pain, hot, and swollen) and more than 50,000 cells/mm^3^ on synovial fluid evaluation, or positive bacterioscopy, or positive synovial fluid culture, or positive blood culture. All patients underwent medial parapatellar arthrotomy for joint cleaning and debridement.

Cases of polyarticular septic arthritis, skeletal immaturity, post-surgical infection, and cases in which no arthrocentesis (joint aspiration) was performed in the initial care were excluded.

The demographic, clinical, surgical, and microbiological variables of the cases were analyzed, through a review of medical records, in order to obtain the following data: Identification: gender, age, and comorbidities associated with immunosuppression (diabetes, chronic kidney disease, smoking, rheumatoid arthritis, alcoholism, substance-related disorders, active cancer, and HIV infection); Laboratory analysis of joint aspiration: global cell count, percentage of neutrophils, bacterioscopy, and synovial fluid culture; Complications: need for surgical reoperation and death.

The equality test of two proportions was applied to analyze the prevalence (relative frequency) of some qualitative covariates. A chi-square test was also used to measure the relationship between positive culture and cell count in the synovial fluid, as well as between the positive cultures for *Staphylococcus aureus* and the positive cultures for the other germs for prediction of reoperation and death. Finally, multivariate logistic regression analysis was applied to identify the independent factors (age, comorbidity, cellularity, and fluid culture) to the need for reoperation and death. Prevalence was estimated for the total valid answers of each variable. To evaluate the objectives of this work, the collected data were grouped into tables and subjected to statistical analysis using the programs SPSS V20, Minitab 16, and Excel Office 2010. A significance level of 0.05 (5%) was defined for this study. The confidence interval was defined as 95% statistical confidence.

## RESULTS

In the period analyzed, 49 cases of septic arthritis of the knee treated surgically in our service were identified, and 38 of them were eligible for this study. A total of five cases with skeletal immaturity and five cases of post-surgical infection were excluded. The age of the patients ranged from 19 to 81 years, with an average of 49.1 years. Men were the most affected, with 29 cases (76.3%). Regarding the presence of comorbidities, 13 patients (34.2%) had no comorbidity, 10 (26.3%) had one, and 15 (39.4%) had two or more. Regarding synovial fluid cellularity, the number of nucleated cells varied from 6,800 to 720,000 cells/mm^3^, and 23 patients (60.5%) had more than 50,000 cells/mm^3^ in the liquid. The percentage of neutrophils ranged from 75% to 99%, being higher than 90% in 32 patients (84.2%).

**Figure 1:**
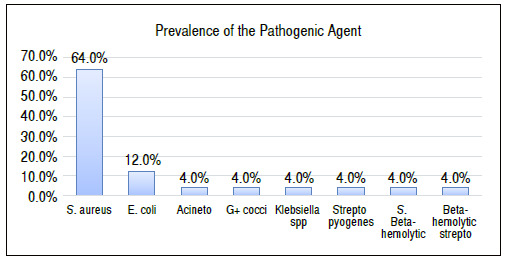
The most frequently identified infectious agent in synovial fluid cultures was *Staphylococcus aureus*.

Bacterioscopy was positive in 45% of the cases, all for gram-positive cocci, and synovial fluid culture was positive in 73% of the cases. As for the pathogens identified, 64% of the infections were caused by *Staphylococcus aureus* (Figure 1).

*S. aureus*: *Staphylococcus aureus*; *E. coli*: *Escherichia coli*; multi-R *Acineto*: Multidrug-Resistant *Acinetobacter*; G+ cocci: Gram-positive cocci; S. Beta-hemolytic: beta-hemolytic *Staphylococcus*; *Strepto pyogenes*: *Streptococcus pyogenes*; Beta-hemolytic *strepto:* beta-hemolytic *Streptococcus*.

Regarding the number of surgical debridements necessary for controlling the infectious process, in 50% of the cases only one surgical approach was performed and in the other 50% at least one more surgical debridement after the initial operation was necessary. In our sample, the average number of debridements to control the infectious process was 2.02. Mortality during hospitalization was 10.5% (four cases).

Higher synovial fluid cell count and higher culture positivity showed no correlation, and 44% of patients with positive synovial fluid culture had less than 50,000 cells/mm^3^ in the synovial fluid (Table 1).

**Table 1:** Correlation between cultures with synovial fluid pathogen identification and global cell count.

	Negative culture		Positive culture		Total		
	N	%	N	%	N	%	
**> 50 thousand/mm** ^3^	7	77.8%	14	56.0%	21	61.8%	p-value 0.249
**≤ 50 thousand/mm** ^3^	2	22.2%	11	44.0%	13	38.2%	
**Total**	9	26.5%	25	73.5%	34	100.0%	

thousand/mm^3^: thousand units per cubic millimeter; N: total number. %: percentage; p-value: probability of significance.

A significant correlation between the need for reoperation and isolation of *S. aureus* in cultures was observed (p = 0.041). We estimated the odds ratio, in which in the analysis with reordering it was 6.00. This value was important because the 95% confidence interval does not contain the value 1.00 in it, indicating that patients with positive cultures for *S. aureus* were six times more likely to require reoperation to joint cleansing and debridement compared to patients with positive cultures for other agents (Table 2).

**Table 2:** Relationship of infections caused by Staphylococcus *aureus* and other agents with the number of reoperations and deaths.

Reoperations	Culture (+) S aureus (n = 16)	Culture (+) other agents (N = 9)	p-value	Odds ratio
	N	N		
Yes	12 (75%)	3 (33.3%)	0.041	6
No	4 (25%)	6 (66.6%)		(1.01 to 35.91)
**Deaths**				
Yes	2 (12.5%)	1 (11.1%)	0.918	1.14
No	14 (87.5%)	8 (88.8%)		(0.09 to 14.68)

N: total number; %: percentage; p-value: probability of significance.

No other isolated factor evaluated can be considered as a risk predictor, since none of them was statistically significant for a higher need for reoperation or higher risk of mortality (Table 3).

**Table 3:** Logistic regression model to evaluate factors associated with higher risk of reoperation and death.

		p-value	Odds ratio 95%CI		
			OR	Inferior Limit	Superior Limit
**Reoperation**	Constant	0.032	0.03		
	Age	0.395	1.02	0.97	1.07
	Comorbidities	0.226	2.9	0.52	16.22
	Cells/mm^3^ > 50,000	0.059	5.44	0.94	31.5
	Liquid culture	0.166	3.76	0.58	24.46
**Death**	Constant	0.998	0		
	Age	0.754	1.01	0.93	1.11
	Comorbidities	0.999			
	Cells/mm^3^ > 50,000	0.998			
	Liquid culture	0.917	1.18	0.05	25.38

OR: Odds ratio; cells/mm^3^: cell count per cubic millimeter; p-value: probability of significance.

## DISCUSSION

In this study, 50% of patients with infectious arthritis required reoperation, the mortality rate of the cases was 10.5%, and the average number of debridements needed to control the infection was 2.02. The presence of positive synovial fluid culture and the cell count in the liquid showed no relationship. Patients with infection caused by *S. aureus* were six times more likely to undergo reoperation compared to patients with positive cultures for other infectious agents.

Risk factors for pyogenic arthritis are the presence of comorbidities such as advanced age, diabetes mellitus, renal failure, and other conditions associated with impaired immunity. ^(^ ^11^ ^)- (^ ^13^ A total of 65.8% of our patients had some type of comorbidity associated with immunosuppression; however, the presence of these comorbidities showed no association with a higher risk for reoperation or death.

The most commonly identified pathogenic agent in cases of septic arthritis of the knee is *Staphylococcus aureus*,^12^ ^), (^ ^15^ and some studies associate the isolation of this agent in cultures with a worse prognosis, with a higher risk of needing reoperations to new cleaning and debridement of the joint. ^(^ ^12^ In our sample, *Staphylococcus aureus* was identified in 64% of positive cultures, being the most prevalent agent and associated with the worst prognosis. In cases in which this pathogen was isolated, there was a greater need for reoperation, with statistical significance, compared to cases in which other germs were identified in cultures. Patients with cultures positive for *Staphylococcus aureus* were six times more likely to require reoperation for joint cleansing and debridement compared to patients with positive cultures for other agents.

The evaluation of synovial fluid is essential for the diagnosis of septic arthritis. In the literature, bacterioscopy is positive in 30 to 50% of cases of infection, ^(^ ^6^ ^), (^ ^7^ ^)^ and in our series it was positive in 44.8% of the cases, hence the importance of evaluating the cytology of the synovial fluid for diagnosis in urgency.

The most commonly used cutoff number for the number of white cells in the synovial fluid in the diagnosis of septic arthritis is a count greater than 50,000 cells/mm^3^.^6^ ^)- (^ ^10^ However, Bell et al. ^(^ ^16^ identified that only 31.2% of patients with positive cultures had white cell counts ≥ 50,000 cells/mm^3^. In our series, 44% of patients with positive synovial fluid culture had less than 50,000 cells/mm^3^ in the synovial fluid. These findings suggest that the cutoff value of more than 50,000 cells/mm^3^ for the diagnosis of septic arthritis may be a very high value. Further studies, however, are needed to elucidate this hypothesis.

Stake et al. ^(^ ^13^ described that high cellularity in the synovial fluid is related to the increase in the rate of reoperation, and an increase of 1,000 cells/mm^3^ is related to a 1% increase in the chance of reoperation. In our study, patients with cell counts ≥ 50,000 cells/mm^3^ tended to present a higher risk of need for reoperation, but without statistical significance (p = 0.059).

The most commonly used options for surgical treatment of septic arthritis of the knee are open or arthroscopic cleansing and debridement. The literature lacks consensus; however, a trend in favor of using the arthroscopic technique is observed. ^(^ ^6^ In our series, we performed the surgical treatment of septic arthritis of the knee by open approach, through arthrotomy. This was due to the unavailability of arthroscopic material for urgent surgical procedure in our service.

The rate of reoperation in cases of septic arthritis of the knee varies, ranging from 0 to 71% in the literature. ^(^ ^1^ ^), (^ ^12^ In our series, reoperation was required in 50% of cases. In the literature, the series of septic arthritis treated arthroscopically tend to have a lower rate of reoperation than the series treated by arthrotomy. In the series of Peres et al., ^(^ ^17^ no case treated by arthroscopy required more than one surgical procedure. Böhler et al. ^(^ ^18^ ^)^ needed reoperation in only 5% of the cases treated by arthroscopy. On the other hand, Johns et al. ^(^ ^19^ ^)^ showed a reoperation rate of 50% in cases treated by arthroscopy, and 71% in cases treated by arthrotomy; the average number of procedures necessary to control the infection was 1.79 in arthroscopic treatment, and 2.42 in open approach. In our series of cases treated by arthrotomy, 2.02 procedures were needed on average to control the infectious process.

The mortality rate of our sample was 10.5%. Ferrand et al., ^(^ ^20^ in a study with 109 patients, had 5.6% of deaths. Balalaud et al. ^(^ ^2^ in a literature review, showed a mortality rate of 3 to 29%.

Our sample, relatively small, represents a limitation of our study. It was a convenience sample based on the number of cases that were admitted to the hospital over the collection period. Additionally, surgical procedures were performed by more than one surgeon, as it is a teaching hospital. However, the use of a standardized technique, medial parapatellar arthrotomy, allowed an effective debridement of the joint.

## CONCLUSION

In 50% of the cases of septic arthritis of the knee of adult individuals, treated with surgical cleaning and open debridement, a new surgical approach was necessary. The average number of debridement was 2.02 and the mortality rate during hospitalization was 10.5%. Patients with cultures positive for *Staphylococcus aureus* were six times more likely to require reoperation compared to patients with positive cultures for other agents.
